# Ablação Septal com Radiofrequência e Uso de Novas Tecnologias em Pacientes com Cardiomiopatia Hipertrófica no Laboratório de Eletrofisiologia

**DOI:** 10.36660/abc.20220143

**Published:** 2022-10-05

**Authors:** Muhieddine Chokr, Marina Mayrink, Pedro Mario Pinto Vandoni, Pedro Vieira Linhares, Italo Bruno dos Santos Sousa, Hélio José Castello, Mauricio Scanavacca

**Affiliations:** 1 Universidade de São Paulo Faculdade de Medicina Hospital das Clinicas São Paulo SP Brasil Universidade de São Paulo Faculdade de Medicina Hospital das Clinicas Instituto do Coração, São Paulo , SP – Brasil; 2 Grupo Angiocardio Hemodinâmica São Paulo SP Brasil Grupo Angiocardio Hemodinâmica – Cardiologia, São Paulo , SP – Brasil

**Keywords:** Ablação por radiofrequência, Cardiomiopatia hipertrófica, Eletrofisiologia

Prezado Editor,

Lemos com grande interesse o artigo “Ablação septal com cateteres e radiofrequência guiada pela ecocardiografia para tratamento de pacientes com cardiomiopatia hipertrófica obstrutiva (CHO): Experiência inicial”, publicado recentemente por Valdigem et al. ^1^ nos Arquivos Brasileiros de Cardiologia.

Nesse estudo, os autores avaliaram os efeitos da ablação endocárdica por radiofrequência (RF) do septo interventricular com redução do gradiente ventrículo-arterial e melhora de classe funcional em 12 pacientes com CHO. Cateteres com pontas sólidas de 8 mm de comprimento foram utilizados para aplicação de RF termo controlada. A intensidade de energia foi de 80 Watts com temperatura máxima de 60 ^0^ C. A região de maior gradiente na via de saída do ventrículo esquerdo foi o alvo para ablação e identificada pelo ecocardiograma transesofágico. Os autores observaram uma redução média dos gradientes obtidos de 96,8±34 mmHg para 36,1±23 mmHg (p=0,0001) no seguimento de 1 ano, com melhora clínica em todos os pacientes da série. Concluíram que a ablação septal com RF é uma estratégia eficaz, segura e uma nova opção para tratamento de pacientes com CHO com gradiente elevados e sintomáticos. Parabenizamos os autores pelos bons resultados ao utilizar tecnologia de fácil acessibilidade e ao trazer novas informações sobre um procedimento ainda em desenvolvimento.

**Figura 1:**
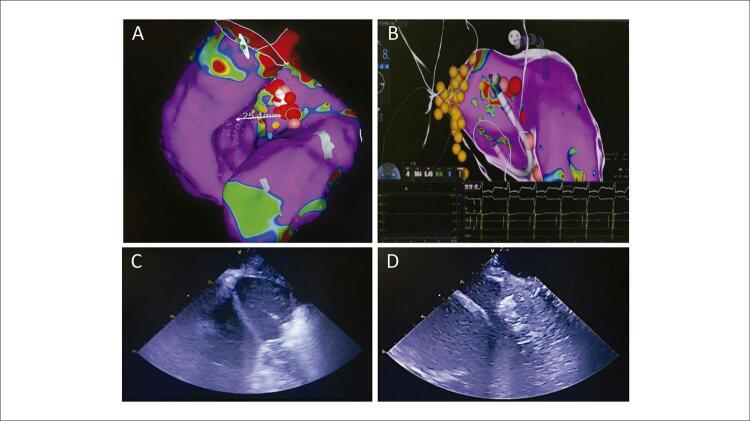
A) Mapa eletroanatômico Carto 3 do ventrículo direito e esquerdo. Pode-se observar o ponto de maior espessamento septal (25 mm). Os pontos em vermelho representam a região onde foi aplicada radiofrequência. B) Início da aplicação de radiofrequência. Os pontos amarelos representam as áreas a serem evitadas em que foi identificado sistema de condução. C) Hiperecogenicidade da região septal, avaliada continuamente com ecocardiografia intracardíaca durante a aplicação de radiofrequência. É possível identificar cateter em alça apoiado sobre a região septal. D) Ao término do procedimento, observado intenso edema na região septal, associado a hiperecogenicidade próximo a via de saída do ventrículo esquerdo.

No período de agosto de 2020 a janeiro de 2021, realizamos ablação com RF do septo interventricular em dois pacientes (homem de 44 anos e mulher de 38 anos de idade) com CHO sintomática, refratários ao tratamento clínico, ambos com seguimento superior a 12 meses. Entretanto, diferentemente da técnica descrita por Valdigem et al., ^1^ utilizamos novas tecnologias de imagem, como mapeamento eletroanatômico (MEA) e o ecocardiograma intracardíaco ( Figura 1 ). O MEA permitiu delimitar a localização do sistema de condução intraventricular e conferiu maior segurança na aplicação de RF (evitar o bloqueio do ramo esquerdo ou atrioventricular total). A construção da geometria pelo MEA dos ventrículos esquerdo e direito também forneceu informação importante na delimitação da área a ser abordada. O ecocardiograma intracardíaco (ICE) permitiu acompanhar a produção das lesões de RF no septo interventricular e a evolução do edema próximo à via de saída do ventrículo esquerdo durante o procedimento, sem a necessidade de um ecocardiografista. Adicionalmente, a ablação com radiofrequência foi otimizada com a utilização de cateteres com ponta irrigada, e as lesões foram controladas pelo software VISITAG SURPOINT (J&J) ^2^ para uniformizar sua profundidade.

O critério de interrupção do procedimento utilizado por Valdigem et al. ^1^ foi a queda aguda de 25% no gradiente ventrículo-arterial. No entanto, alguns autores sugerem que a ablação septal excessiva para atingir esses índices agudamente pode provocar aumento paradoxal e agudo do gradiente com risco de congestão pulmonar importante após a ablação. ^3^ Nossa impressão é que o uso de uma estratégia puramente anatômica, com aplicações septais logo acima do ramo esquerdo, tendo como alvo um *Ablation Index* entre 600 e 700, usando cateter irrigado de 3,5 mm (50 Watts e 43 ^0^ C) e avaliação contínua do edema da via de saída do ventrículo esquerdo com o ICE, pode tornar o procedimento mais seguro.

As diversas séries publicadas até o momento não valorizam o gradiente imediato sugerindo que o maior benefício na redução do gradiente ocorre entre 9 e 12 meses da ablação. ^4 , 5^ Nossos pacientes tiveram uma redução significativa do gradiente intraventricular, com redução média de 91±22 mmHg para 27±14 mmHg cerca de 12 meses após o procedimento índice, e redução no primeiro dia de pós-operatório de 22±6 mmHg, ambos com melhora significativa dos sintomas e atualmente em classe funcional II. A utilização de cateter irrigado, permite a realização de lesões mais previsíveis, mas pode contribuir com quadros de congestão pulmonar como descrito pelos autores. A utilização simultânea do ICE para acompanhar as aplicações de RF também pode evitar a ocorrência de *“Stem Pops”* , fato comum em aplicações prolongadas e com alta energia. Adicionalmente, o ecocardiograma intracardíaco auxilia na monitoração do risco de aplicações excessivas ao acompanhar a formação de edema septal. Apesar disso, um dos nossos pacientes apresentou quadro de congestão pulmonar imediatamente após a ablação, que foi resolvido com uso de diuréticos e ventilação não invasiva. Tanto a utilização de cateter irrigado como o edema importante na via de saída podem ter contribuído para o quadro apresentado pela paciente. Novos estudos são necessários a fim de comparar diferentes técnicas bem como padronizar qual seria a forma ideal de criar as lesões, que minimizem o risco de aumentos agudos de gradiente ventrículo-arterial após a ablação.
