# Supplementary material for: Ablação Septal com Radiofrequência e Uso de Novas Tecnologias em Pacientes com Cardiomiopatia Hipertrófica no Laboratório de Eletrofisiologia
Source: Arq Bras Cardiol. 2022 Oct 5;119(4):635–7. [Article in Portuguese] doi: 10.36660/abc.20220143 (PMC9563876; doi:10.36660/abc.20220143)
Supplement: Translation

# Translation: Septal Ablation with Radiofrequency and the Use of New Technologies in Patients with Hypertrophic Cardiomyopathy in an Electrophysiology Laboratory

**Authors:** Muhieddine Chokr, Marina Mayrink, Pedro Mario Pinto Vandoni, Pedro Vieira Linhares, Italo Bruno dos Santos Sousa, Hélio José Castello, Mauricio Scanavacca

**Affiliations:** 1 Brazil Universidade de São Paulo Faculdade de Medicina Hospital das Clinicas Instituto do Coração, São Paulo, SP – Brazil; 2 Brazil Grupo Angiocardio Hemodinâmica – Cardiologia, São Paulo, SP – Brazil

**Keywords:** Radiofrequency Ablation, Hypertrophic Cardiomyopathy, Electrophysiology

Dear Editor,

We have read, with great interest, the article “Septal ablation with catheters and radiofrequency guided by echocardiography for treatment in patients with obstructive hypertrophic cardiomyopathy (OHC): First experience”, published recently by Valdigem et al.^1^ in the journal *Arquivos Brasileiros de Cardiologia* .

In this study, the authors evaluated the effects of endocardial ablation by radiofrequency (RF) of the interventricular septum with reduction of the ventricular-arterial gradient and improvement in functional class in 12 patients with OHC. Catheters with solid 8-mm tips were used to apply the thermo-controlled RF. The energy intensity was of 80 Watts, with a maximum temperature of 60ºC. The target for ablation was the region with the highest gradient in the left ventricle outflow tract and was identified by the transesophageal echocardiogram. The authors observed an average reduction of the gradients obtained from 96.8±34 mmHg to 36.1±23 mmHg (p=0.0001) during a 1-year follow-up, with a clinical improvement in all patients of the series. They concluded that the septal ablation with RF is an effective, safe strategy and represents a new option to treat OHC patients with high and symptomatic gradients. We would like to congratulate the authors for their fine results in using a technological device that is of easy access, as well as for bringing new and relevant information about the procedure, which is still under development.

From August 2020 to January 2021, our team conducted an ablation with RF of the interventricular septum in two patients (a 44-year-old man and a 38-year-old woman) with symptomatic OHC, who were refractory to clinical treatment, both undergoing follow-up for more than 12 months. However, in contrast to the technique described by Valdigem et al.,^1^ we used new imaging techniques, including electroanatomic mapping (EAM) and intracardiac echocardiography (ICE) ( Figure 1 ). The EAM allowed us to define the localization of the intraventricular conduction system and conferred greater safety in the application of RF (avoiding the left bundle branch block or complete atrioventricular block). The geometric construction produced by the EAM of the left and right ventricles also provided important information on the definition of the area to be treated. The ICE allowed us to follow the production of the lesions of RF in the interventricular septum and the evolution of the edema near the left ventricle outflow track during the procedure, without the need for an echocardiographer. Additionally, the ablation with radiofrequency was optimized with the use of irrigated-tipped catheters, and the lesions were controlled by the VISITAG SURPOINT (J&J)^2^ software in order to standardize its depth.

**Figure 1:** Carto 3-guided electroanatomic maps of the right and left ventricle. This picture shows the point with the largest septal thickness (25 mm). The points in red represent the region where the radiofrequency was applied. B) Beginning of the application of radiofrequency. The yellow points represent the areas to be avoided in which the conduction system was identified. C) Hyperechogenicity of the septal region, evaluated continually using the intracardiac echocardiogram during the application of radiofrequency. It is possible to identify the catheter in the strap resting on the septal region. D) At the end of the procedure, an intense edema is observed in the septal region, associated with hyperechogenicity near the left ventricle outflow tract.

The procedure’s interruption criteria used by Valdigem et al.^1^ was an acute 25% drop in the ventricular-arterial gradient. However, some authors suggest that the excessive septal ablation to reach these indexes can acutely provoke a paradoxical and acute increase of the gradient with the risk of significant pulmonary congestion after the ablation.^3^ Our impression is that the use of a purely anatomic strategy, with septal applications directly above the left branch, with an Ablation Index target of between 600 and 700, using an 3.5-mm irrigated catheter (50 Watts and 43ºC) and a continuous evaluation of the edema of the left ventricle outflow tract with the ICE, may well make the procedure safer.

The wide range of series published to date do not give value to the immediate gradient, suggesting that the greatest benefit in the reduction of the gradient occurs between 9 and 12 months of ablation.^4 , 5^ Our patients witnessed a significant reduction of the interventricular gradient, with an average drop from 91±22 mmHg to 27±14 mmHg, approximately 12 months after the initial procedure, and a further reduction in the first post-operative day of 22±6 mmHg, both with a significant improvement in the symptoms. The patient is currently at the functional class II level. The use of an irrigated catheter can cause more predictable lesions, but it can also contribute in treating pulmonary congestion, as described by the authors. The simultaneous use of the ICE to follow up on the RF applications can also prevent the occurrence of *“Stem Pops”* , a common fact in prolonged applications and with high energy. Additionally, the intracardiac echocardiogram aids in monitoring the risk of excessive applications by accompanying the formation of a septal edema. Nevertheless, one of our patients presented a medical condition of immediate pulmonary congestion after the ablation, which was resolved with the use of diuretics and non-invasive ventilation. Both the use of the irrigated catheter as well as the significant edema in the outflow tract may have contributed to the medical condition presented by the patient. Further studies are warranted in order to compare different techniques, as well as to standardize what would be the ideal means through which to create the lesions, which minimize the risk of acute increases in the ventricular-arterial gradient after the ablation.
