# Supplementary material for: Ablação Septal com Radiofrequência e Uso de Novas Tecnologias em Pacientes com Cardiomiopatia Hipertrófica no Laboratório de Eletrofisiologia
Source: Arq Bras Cardiol. 2022 Oct 5;119(4):635–7. [Article in Portuguese] doi: 10.36660/abc.20220143 (PMC9563876; doi:10.36660/abc.20220143)
Supplement: Related texts

# Reply: Carta-resposta

**Authors:** Bruno Valdigem, Andrea de Andrade Vilela, Antonio Tito Paladino, David Le Bihan

Parabéns pelo interesse no procedimento,^1^ acreditamos que novos protocolos de ablação e em especial dos cuidados de pós-operatório devem ser estimulados, visto que mesmo a forma de liberação de energia e a localização do ponto de aplicação da radiofrequência não são um consenso entre os autores (note que Lawrenz em publicação de agosto de 2021, apresentou dados com aplicação bilateral e apenas em septo direito).^2^

Após revisão cuidadosa dos primeiros 40 casos realizados por nosso grupo (publicação pendente) pudemos observar que diferenças relacionadas a morfologia do septo interventricular podem ser importantes na escolha do local de início da ablação. Septos com hipertrofia mais homogênea tendem a apresentar migração do gradiente no sentido apical. Assim, ablação guiada apenas pela anatomia pode resultar em lesões mais extensas sem necessidade. Também podem ocorrer gradientes medioventriculares residuais que implicariam numa segunda abordagem.

Outro benefício do uso do ecocardiograma transesofágico (ETE) seria a localização do ponto de real início do gradiente. Não raramente, o gradiente se inicia mais na região apical que do que na região mais espessa do septo, marcado pelo ponto de maior *aliasing* , evidenciado pelo mapeamento de fluxo a cores ao ecocardiograma ( Figura 1 ). O ETE apresenta imagens com melhor visualização da via de saída do ventrículo esquerdo do que o ecocardiograma intracardíaco, informação que é extremamente importante para adequado alinhamento do cateter.

**Figura 1:** ETE intraprocedimento com e sem color (a 150°): avaliação da valva mitral, identificação do SAM e sua localização. Nessa angulação conseguimos identificar mais facilmente o local de maior aceleração de fluxo e o grau do refluxo mitral.

A avaliação ecocardiográfica diferencia as curvas de obstruções valvares (na estenose aórtica) da subvalvar, por meio da imagem espectral do Doppler contínuo ( Figura 2 ). Tal informação é fundamental nos casos de estenoses sequenciais, como ocorre nos casos de ablação septal por radiofrequência pré-TAVI (em que esse método tem demonstrado um papel promissor).

**Figura 2:** Registro do Doppler contínuo com as curvas relacionadas à estenose aórtica e à obstrução subvalvar.

Adicionalmente, o ETE fornece informações sobre estruturas adjacentes ao ponto de aplicação da radiofrequência, permitindo a pronta identificação de intercorrências possíveis como lesões em cordas tendíneas, ou cúspide anterior da valva mitral.

Um dos parâmetros indicadores de sucesso no intraprocedimento é a redução do refluxo mitral, decorrente da redução no deslocamento sistólico anterior do aparato valvar mitral devido à redução da obstrução na via de saída do ventrículo esquerdo.

Mais uma vez parabenizamos os autores pelas suas considerações e pelo esforço em simplificar o procedimento e aumentar sua propagação. Colocamo-nos à disposição para análise conjunta de dados e trocas de experiências.

## References

[1] Chokr M, Mayrink M, Vandoni PMP, Linhares PV, Sousa IBS, Castello HJ (2022). Septal Ablation with Radiofrequency and the Use of New Technologies in Patients with Hypertrophic Cardiomyopathy in an Electrophysiology Laboratory. Arq Bras Cardiol.

[2] Lawrenz T, Lawin D, Radke K, Stellbrink C (2021). Acute and Chronic Effects of Endocardial Radiofrequency Ablation of Septal Hypertrophy in HOCM. J Cardiovasc Electrophysiol.

# Reply

Congratulations for your interest in the procedure,^1^ we believe that new protocols for ablation and especially for post-operative care should be encouraged, given that even the way of releasing the energy and the localization of the point where the radiofrequency is applied are not a consensus among authors (note that Lawrenz, in a publication in August 2021, presented data with a bilateral application and only in the right septum).^2^

After a careful review of the first 40 cases carried out by our research group (publication pending), we observed that differences related to the morphology of the interventricular septum can be significant in the choice of the location to begin the ablation. More homogeneous hypertrophic septa tend to present a migration from the gradient in the apical direction. Thus, the ablation guided only by the anatomy can result in more extensive lesions unnecessarily. Moreover, residual medioventricular gradients can also occur, which would require a second approach.

Another benefit for the use of the transesophageal echocardiogram (TEE) would be the localization of the real point where the gradient began. More often, the gradient begins in the apical region than in the thicker region of the septum, marked by the point of greater aliasing, illustrated by the color flow mapping echocardiogram ( Figure 1 ). The TEE presents images with a better visualization of the left ventricle outflow tract than does the intracardiac echocardiogram, information that is of utmost importance for the adequate alignment of the catheter.

**Figure 1:** Intra-procedure TEE with and without color (at 150°): evaluation of the mitral valve, identification of the SAM and its localization. From this angle, we were able to more easily identify the location of greater acceleration of the flow and the degree of mitral valve regurgitation.

The echocardiographic evaluation differentiates the shapes of valvular obstructions (in aortic stenosis) from the subvalvar obstructions, through the spectral image of the Continuous Doppler ( Figure 2 ). This information is essential in cases of sequential stenosis, as occurs in the cases of septal ablation by pre-TAVI radiofrequency (in which this method has shown a promising role).

**Figure 2:** Continuous Doppler image with the shapes related to aortic stenosis and subvalvar obstruction

Furthermore, the TEE provides information about the adjacent structures, including the application of radiofrequency, enabling the ready identification of possible complications, such as lesions in tendinous cords, or the anterior cusp of the mitral valve.

One of the main parameters of success in the intra-procedure is the reduction in mitral regurgitation, resulting from the reduction in the systolic anterior motion (SAM) of the mitral valve due to the reduction in the obstruction of the left ventricle outflow tract.

Once again, we would like to congratulate the authors for their considerations and for their efforts to simplify the procedure and increase its diffusion. If it should be of your interest, we are willing to participate in joint analyses and sharing of experiences.
